# Establishment and characterization of induced pluripotent stem cells (iPSCs) from central nervous system lupus erythematosus

**DOI:** 10.1111/jcmm.14598

**Published:** 2019-09-19

**Authors:** Maria Teresa De Angelis, Gianluca Santamaria, Elvira Immacolata Parrotta, Stefania Scalise, Michela Lo Conte, Sara Gasparini, Edoardo Ferlazzo, Umberto Aguglia, Clara Ciampi, Antonella Sgura, Giovanni Cuda

**Affiliations:** ^1^ Department of Experimental and Clinical Medicine, Stem Cell Laboratory, Research Center for Advanced Biochemistry and Molecular Biology “Magna Græcia” University Catanzaro Italy; ^2^ Department of Medical and Surgical Sciences “Magna Græcia” University Catanzaro Italy; ^3^ Regional Epilepsy Centre Great Metropolitan Hospital Reggio Calabria Italy; ^4^ Department of Science University of Rome “ Roma Tre” Rome Italy

**Keywords:** biomarkers, central nervous system‐systemic lupus erythematosus, induced pluripotent stem cells

## Abstract

Involvement of the central nervous system (CNS) is an uncommon feature in systemic lupus erythematosus (SLE), making diagnosis rather difficult and challenging due to the poor specificity of neuropathic symptoms and neurological symptoms. In this work, we used human‐induced pluripotent stem cells (hiPSCs) derived from CNS‐SLE patient, with the aim to dissect the molecular insights underlying the disease by gene expression analysis and modulation of implicated pathways. CNS‐SLE‐derived hiPSCs allowed us to provide evidence of Erk and Akt pathways involvement and to identify a novel cohort of potential biomarkers, namely *CHCHD2*, *IDO1*, *S100A10*, *EPHA4* and *LEFTY1*, never reported so far. We further extended the study analysing a panel of oxidative stress‐related miRNAs and demonstrated, under normal or stress conditions, a strong dysregulation of several miRNAs in CNS‐SLE‐derived compared to control hiPSCs. In conclusion, we provide evidence that iPSCs reprogrammed from CNS‐SLE patient are a powerful useful tool to investigate the molecular mechanisms underlying the disease and to eventually develop innovative therapeutic approaches.

## 1 INTRODUCTION

Systemic lupus erythematosus is a prototype of inflammatory autoimmune disease characterized by the production of autoantibodies against nuclear components.^1^ Among the affected organs, there is the central nervous system. CNS‐SLE shows a wide spectrum of clinical manifestations, which makes it difficult to diagnose. It is not uncommon that SLE is diagnosed after patients are treated for a neurological event.^2^ The heterogeneous nature of SLE suggests that a number of factors play a role in generating the autoantigens associated with the disease. SLE patients show accumulation of apoptotic cells and debris in T lymphocytes, germinal centres (GC), bone marrow and epidermis, probably due to a defective apoptotic clearance activity.^3^, ^4^ Indeed, an impaired removal of apoptotic blebs may lead to an accumulation of apoptotic cells and subsequent release of apoptosis‐ modified nuclear structures (non‐self antigens) induces an immunogenic response.^5^, ^6^, ^7^

Apoptotic events may be caused, among others, by excessive reactive oxygen species (ROS) production. A prolonged interaction between ROS and nuclear debris generates, in turn, additional neo‐epitopes stimulating a broad‐spectrum of autoantibodies.^8^, ^9^ The noxious effects of ROS cause oxidative modifications of lipids, proteins and DNA, which positively correlate with organ damage and severity of SLE.^10^ Nowadays, SLE patients lack effective therapies; current treatments are merely based upon broad‐spectrum immune suppressive regimes. Recently, many efforts have been directed to identify novel biomarkers for diagnosis and prevention of SLE.^11^, ^12^, ^13^ However, the molecular basis and pathogenesis are still far away from complete understanding.

We aimed at deciphering the pathogenesis of CNS‐SLE using fibroblasts‐derived iPSCs obtained from dermal biopsy of a SLE patient (hiPSCs‐SLE) bearing neurological symptoms. To identify novel biomarkers and to investigate on potentially disrupted molecular pathways involved in SLE disease, gene expression profiling of hiPSCs‐SLE and two controls hiPSCs (‐F and ‐L) was performed. This approach not only revealed, already at the pluripotent state, several common markers with another reprogrammed cell line derived from SLE patient without neurological symptoms,^14^ but it was also able to uncover significant differences for markers that may be a distinct trait of SLE patient with implications in the nervous system. In this study, we provide evidence of the implication of Erk and Akt pathways in SLE pathogenesis. Although a correlation between oxidative stress and the progression of SLE has been widely demonstrated, the molecular mechanisms of hydrogen peroxide (H_2_O_2_) injury on hiPSCs‐SLE remain to be further investigated. H_2_O_2_ contributes to oxidative stress by inducing considerable damage in the cellular environment. Antioxidant agents, such as ascorbic acid (AA), are important in counteracting these oxidative effects.^15^ Finally, the potential cytotoxic effect of H_2_O_2_ and the antioxidative function of AA in SLE‐hiPS and controls hiPS cell lines were investigated with a peculiar focus to the involvement of oxidative stress‐related miRNAs.

## 2 MATERIALS AND METHODS

### 2.1 iPSC generation and culture procedures

A female patient with CNS‐SLE Disease Activity Index (SLEDAI) 8 and two healthy subjects were recruited. Skin fibroblasts from the SLE patient were obtained and reprogrammed to generate iPSCs. Fibroblasts were infected by Sendai virus (MOI = 5) containing four reprogramming factors (*c‐MYC*, *KLF4*, *SOX2* and *OCT4*) (Cytotune 2.0; Thermo Fisher Scientific). iPS clones were isolated and expanded upon emergence onto feeder‐free Matrigel (BD Biosciences)‐coated plates and three independent clones of hiPSCs‐SLE were considered as biological replicates. Additionally, female hiPSCs derived from T lymphocytes (hiPSCs‐L) and male hiPSCs derived from skin fibroblasts (hiPSCs‐F) were generated and characterized in our laboratory.^16^, ^17^ All experiments using healthy hiPSCs were performed in independent experiments to obtain biological replicates. The human iPSC lines were grown in mTeSR1 medium (StemCell Technologies) and passaged as small colonies on Matrigel‐coated plates.

To investigate signalling pathways involved in SLE pathology, the specific inhibitors PD0325901 (5 µmol/L—Selleckchem) or LY294002 (20 mmol/L—Merck Millipore) targeting the Mek/Erk or PI3K/Akt signalling pathways, respectively, were added in mTeSR1 medium for 5 hours. To perform oxidative stress and verify the antioxidant role of AA on hiPSCs, cells untreated or pre‐treated for 24 hours with 1 mmol/L AA (Sigma Aldrich) were incubated with 0.2 mmol/L H_2_O_2_ for 4 hours. After treatments, cells were harvested immediately for RT‐qPCR and Western blot analysis.

### 2.2 Alkaline phosphatase (AP) staining

hiPSCs were washed with PBS and fixed with 4% paraformaldehyde (PFA) solution and then rinsed with PBS three times. AP staining was performed using 1‐Step NBT/BCIP (Thermo Fisher Scientific) until the desired colour develops. The reaction was stopped by rinsing well in phosphate‐buffered saline (PBS).

### 2.3 Immunofluorescence

Fixed cells with 4% PFA were permeabilized with PBS containing 0.3% Triton X‐100 and then blocked with 3% bovine serum albumin (BSA) (Sigma Aldrich). Human anti‐Nanog (1:1000; rabbit polyclonal, PA1‐097; Thermo Fisher Scientific), anti‐Oct4 (1:400 mouse monoclonal, 60 093; Stem Cell Technologies), anti‐brachyury (1:20 goat polyclonal, AF2085; R&D systems), anti‐Sox17 (1:20 goat polyclonal, AF1924; R&D systems) and anti‐Otx2 (1:20 goat polyclonal, AF1979; R&D systems) primary antibodies diluted in 3% BSA were incubated for 3 hours. The following secondary antibodies: goat anti‐mouse Alexa‐Fluor‐647 (A‐21235; Life Technologies), donkey anti‐goat Alexa Fluor‐594 (A‐11058; Life Technologies) and goat anti‐rabbit Alexa‐Fluor‐488 (A‐11008; Life Technologies) were used for detection. 4',6‐diamidino‐2‐phenylindole (DAPI) was used to counterstain the nuclei. The images were acquired with Leica DMi8 inverted microscope, filter cubes and software from Leica microsystems.

### 2.4 Chromosome spreads

Chromosome spreads were obtained after 16 hours incubation in 0.1 μg/mL nocodazole (Merck KGaA) and dimethyl sulfoxide (DMSO) (Fluka Analytical). Spreads were prepared by a standard procedure consisting of treatment with a buffered hypotonic solution 25 minutes at 37°C, followed by 3 washes in freshly prepared Carnoy's solution (3:1 v/v methanol/acetic acid).^18^ Cells were then dropped onto slides, air dried and utilized for m‐FISH analysis.

### 2.5 Multicolour fluorescent in situ hybridization (m‐FISH)

The m‐FISH protocol was performed accordingly to the procedure described by Berardinelli F et al.^19^ Briefly, fixed cells were denatured in 0.07N NaOH; meanwhile, the probe mix (24XCyte Human Multicolour FISH Probe Kit—MetaSystems, Altlussheim, Germany) was denatured using a MJ mini personal thermal cycler (Bio‐Rad laboratories) with the following program: 5 minutes at 75°C, 30 seconds at 10°C and 30 minutes at 37°C. The slides were rinsed in a graded ethanol series, and probe and slides were hybridized in a humidified chamber at 37°C for 48 hours and counterstained with DAPI. Finally, metaphases were visualized and captured using an Axio‐Imager M1 microscope (Zeiss). The karyotyping and cytogenetic analysis of each single chromosome was performed using the ISIS software (MetaSystems).

Each chromosome of a metaphase spread was examined based on its unique fluorochrome profile.

### 2.6 Embryoid body (EB) formation assay

Single‐cell suspensions from hiPSC colonies were cultured with mTeSR1 medium supplemented with 10 μmol/L of the Rho‐kinase inhibitor Y‐27632 (Selleckchem) in ultra‐low attachment plate (Corning) for 3 days to obtain cell aggregation. At day 8, EBs were cultured in medium consisting of DMEM‐F12 supplemented with 20% knockout serum replacement (KSR), 2 mmol/L L‐glutamine, 0.1 mmol/L non‐essential amino acids, 0.1 mmol/L 2‐mercaptoethanol, and 0.5% penicillin and streptomycin on gelatin‐coated plates for 10 days.

### 2.7 MTT assay

hiPSCs were seeded into a 96‐well plate at a density of 5x10^3^ cells per well. When the cells were 80%‐90% confluent, were treated and analysed. To assess cell viability after treatment with AA, two concentrations of AA (1 and 10 mmol/L) for 24 hours were tested. To determine which H_2_O_2_ dosage was associated with initiation of cell death and relative protective effect of AA, different concentrations of H_2_O_2_ (0.1, 0.2 and 0.4 mmol/L) were used for 4 hours on hiPSCs untreated or pre‐treated with AA 1 mmol/L.

0.5 mg/mL 3‐[4,5‐dimethylthiazol‐2‐yl]‐2,5‐diphenyl‐tetrazolium bromide (MTT) constituted in culture media were added 1h before stopping the treatments. Subsequently, MTT solution was replaced by 2‐propanol (Sigma Aldrich) to dissolve the purple formazan crystals. The plate was then agitated for 10 minutes for solubilization and the absorbance was measured at 570 nm in Multiskan microplate reader (Thermo Fisher Scientific).

### 2.8 RNA isolation and reverse transcription and quantitative real‐time PCR (RT‐qPCR)

Total RNA was extracted from cells using TRIzol reagent (Invitrogen) and reverse‐transcribed using High‐Capacity cDNA Reverse Transcription Kit (Applied Biosystems) according to manufacturer's instructions. Primers were designed using Primer3 software and synthesized by Eurofins Genomics. Oligonucleotide sequences are listed in Table 1.

**Table 1:** List of oligo pair sequences used for RT‐PCR and RT‐qPCR

Gene	Forward primer sequence (5ʹ‐3ʹ)	Reverse primer sequence (5ʹ‐3ʹ)
*CCL26*	AGTCTCCACCTTGGAACTG	AGTCACAATTGTTTCGGAGTT
*CHCHD2*	GGAAGTAATGCTGAGCCTGC	ACCCTCACAGAGCTTGATGT
*c‐MYC Tg*	TAACTGACTAGCAGGCTTGTCG	TCCACATACAGTCCTGGATGATGATG
*EPHA4*	TTTGTCATCAGCCGGAGACG	CTCTCGCACTGCTTGGTTGG
*GAPDH*	TCCTCTGACTTCAACAGCGA	GGGTCTTACTCCTTGGAGGC
*GATA4*	GGCCTGTCATCTCACTACGG	ATGGCCAGACATCGCACT
*HAND1*	CCAGCTACATCGCCTACCTG	CCGGTGCGTCCTTTAATCCT
*IDO1*	AGTTCTGGGATGCATCACCA	ACTGCAGTCTCCATCACGAA
*IGFBP5*	TCAACGAAAAGAGCTACCGC	TTCTGCGGTCCTTCTTCACT
*KLF4 Tg*	TTCCTGCATGCCAGAGGAGCCC	AATGTATCGAAGGTGCTCAA
KOS *Tg*	ATGCACCGCTACGACGTGAGCGC	ACCTTGACAATCCTGATGTGG
*LEFTY1*	CTCTTCCAGGAGCCGGTC	CTGCCAGAAGTTCACGGC
*NANOG*	TGCAAGAACTCTCCAACATCCT	ATTGCTATTCTTCGGCCAGTT
*OCT4*	GGAGGAAGCTGACAACAATGAA	GGCCTGCACGAGGGTTT
*PAX6*	CAGCTTCACCATGGCAAATAA	ATCATAACTCCGCCCATTCA
*REX1*	GTGTGAACAGAACAGAAGAGGC	CTGGTGTCTTGTCTTTGCCC
*S100A10*	AACAAAGGAGGACCTGAGAGTAC	CTTTGCCATCTCTACACTGGTCC
SeV *Tg*	GGATCACTAGGTGATATCGAGC	ACCAGACAAGAGTTTAAGAGATATGTATC
*SOX2*	GGGAAATGGGAGGGGTGCAAAAGAGG	TTGCGTGAGTGTGGATGGGATTGGTG

Abbreviations: Tg, transgene; KOS, *KLF4*, *OCT4*, *SOX2;* SeV, Sendai virus.

qPCR assay was performed using Fast SYBR Green PCR Master Mix (Applied Biosystems) on a StepOnePlus real‐time PCR system (Applied Biosystems). C*_t_* value for each target gene was determined using StepOne software v2.3 (Applied Biosystems). The glyceraldehyde‐3‐phosphate dehydrogenase (*GAPDH*) Ct value was used for the internal control.

miRNA isolation was performed using mirVANA miRNA isolation kit (Thermo Fisher Scientific), and samples were processed with the miRCURY LNA Universal RT microRNA PCR kit (Qiagen) to conduct first‐strand cDNA synthesis and real‐time PCR amplification on Pick‐&‐Mix microRNA PCR Panel according to the manufacturer's instructions. For microRNA quantitative analyses, U6 was used as an internal control and normalized data were further corrected to miR‐103a. The primers used for qPCR were obtained from the Exiqon Services (Qiagen). The quantification of the mRNA or miRNA target genes was calculated using the 2‐ΔΔCt method.^20^ Fold changes were calculated based on the average of three different biological samples.

### 2.9 Western blot

Cell lysates were prepared as previously described.^16^ 25 ug of proteins were loaded and separated by 12% SDS‐PAGE, transferred onto a nitrocellulose membrane (Bio‐Rad). Membranes were hybridized with the following primary antibodies: phospho‐Akt Ser473 (9271; Cell Signalling), Akt1 (2967; Cell Signalling), phospho‐Erk1/2 Thr202/Tyr204 (9101; Cell Signalling), Erk1/2 (9107; Cell Signalling), c‐Fos (2250; Cell Signalling), Caspase‐3 (9668; Cell Signalling), Cleaved Caspase‐9 (9505; Cell Signalling), PARP (9532; Cell Signalling). Subsequently, the membranes were rinsed in Tris‐buffered saline‐0.1% Tween 20 (TBST) and incubated with horseradish peroxidase (HRP)‐conjugated secondary antibodies (Jackson ImmunoResearch). Immunoreactive protein bands were probed using an enhanced chemiluminescence detection system (Bio‐Rad) and acquired with UVITEC Imaging Systems. Actin (sc‐1616, Santa Cruz) antibody was used as loading control.

### 2.10 RNA microarray and PluriTest analysis

RNA was extracted using the RNeasy mini kit (Qiagen) according to manufacturer's procedures. The RNA integrity was evaluated by electrophoresis with a 2200 TapeStation instrument (Agilent Technologies).

Samples with RNA integrity number (RIN) >8 were selected and amplified with the Illumina TotalPrep RNA amplification Kit (Ambion, Life Technologies). cRNA was hybridized on Human HT‐12 v4 BeadChip (Illumina) and scanned with iScan System (Illumina). Raw data were processed and analysed using the PluriTest algorithm.^21^

### 2.11 Bioinformatic analysis

Illumina HumanHT‐12‐V4 raw intensity data were imported in R statistical environment using *limma* package^22^ for control probes following background subtraction, quantile normalization and log_2_ transformation signal values (File S1). Moderated *t* test analysis with Benjamini and Hochberg (B‐H) multiple testing correction was utilized to obtain genes whose fold change between comparisons was ≥|1.5| with a *q‐*value cut‐off of ≤0.05.^23^ To improve the bioinformatic analysis, we next consulted the integrated analysis of mRNA online data setting the same thresholds. A graphical representation of common differentially expressed genes (DEGs) was made using *VennDiagram* package.^24^ Ingenuity Pathways Analysis (IPA) (Ingenuity Systems Inc) was used to identify canonical signalling pathways and construct functional interaction of selected networks. The differential gene list was uploaded into the IPA server and statistics for functional analysis was carried out by Fischer's exact test. The ratio between the number of identified genes in a particular pathway and the total number of genes that make up that pathway provides an estimation of the extent of pathway involvement. The enriched canonical pathways were ranked by − log (*P*‐value). Moreover, functional analysis of miRNA target genes was performed using IPA ‘microRNA Target Filter’ program. miRNA targets that were associated with biological functions, canonical pathways and molecular networks related to SLE in the Ingenuity Pathways Knowledge Base were highlighted in the circos plot using *GOplot* package.^25^

### 2.12 Statistical analysis

All analyses were performed using GraphPad Prism version 6. Data were obtained from 3 biological replicates, and all values were presented as mean ± standard error of the mean (SEM). Statistical analysis was performed by unpaired two‐tailed *t* test between controls and experimental groups, *P‐*values of less than 0.05 were considered statistically significant.

## 3 RESULTS

### 3.1 Generation and characterization of hiPSCs‐SLE

In this study, a female patient with CNS‐SLE and two healthy controls were recruited after achieving informed consent.

**Figure 1:**
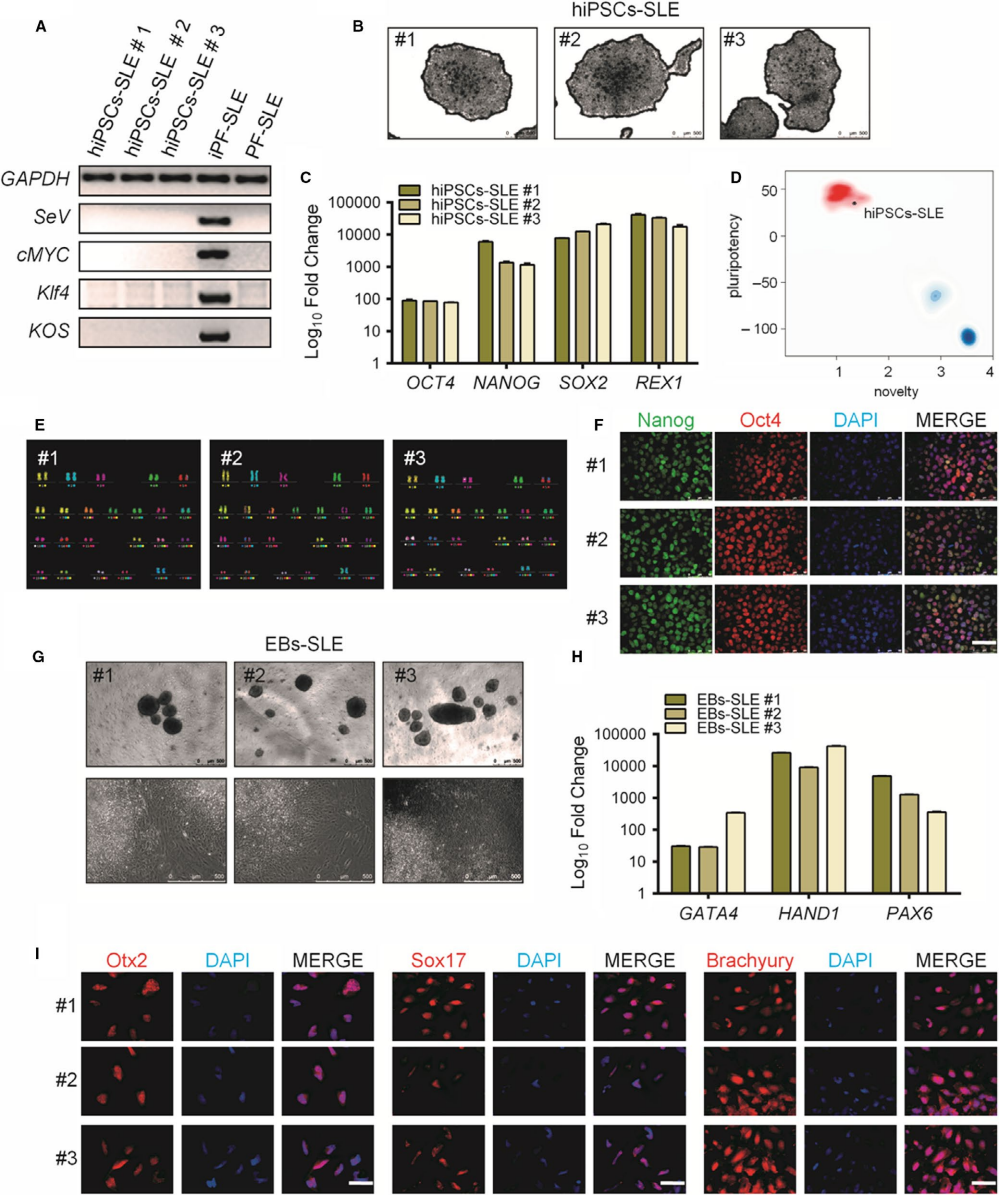
Generation and characterization of systemic lupus erythematosus (SLE)‐specific human‐induced pluripotent stem cells (hiPSCs). (A) Dermal fibroblasts derived from patient with SLE were reprogrammed into iPSCs using Sendai virus vectors and three clones (#1, #2 and #3) were characterized. RT‐PCR confirms the loss of transgenes in hiPSCs‐SLE (lanes 1, 2 and 3), presence (lane 4) in infected fibroblasts (ipF‐SLE) and absence of Sendai viral transgenes in parental fibroblasts (pF‐SLE) (lane 5). Full‐length gels are presented in File S2. (B) The hiPSCs‐SLE colonies expressed alkaline phosphatase. Scale bar, 500 μm. (C) RT‐qPCR analysis of pluripotency genes *OCT4*, *NANOG*, *SOX2* and *REX1* was performed in fibroblasts and in hiPSCs derived from patient with SLE. All expression values are normalized to *GAPDH* and relative donor fibroblasts. Data are mean ± SEM and all statistical analysis was made between hiPSCs‐SLE clones and relative fibroblasts by Student's *t* test showing *P*‐values ≤ .05 in each comparison. (D) PluriTest assays combines novelty score (blue) on x‐axis and pluripotency score (red) on y‐axis. hiPSCs‐SLE localize in the red cloud suggesting the empirical distribution of pluripotent cells compared to non‐pluripotent blue cloud. (E) Representative images of M‐FISH staining show normal karyotypes of hiPSCs‐SLE clones. (F) Immunofluorescence analysis of pluripotent stem cell markers Nanog (green), Oct4 (red) and co‐staining with DAPI (blue) in hiPSCs‐SLE. Scale bar, 50 μm. (G) Representative images of floating and adherent EBs derived from hiPSCs‐SLE at differentiation day 8 and 18, respectively. Scale bar, 500 μm. (H) RT‐qPCR results confirm the capability of hiPSCs‐SLE to differentiate into all three germ layers. The expression levels of *GATA4*, *HAND1* and *PAX6* in EBs are relative to undifferentiated hiPSCs. All expression values are normalized to *GAPDH* and relative hiPSCs. Data are mean ± SEM and all statistical analysis was made between EBs‐SLE and relative hiPSCs‐SLE clones by Student's *t* test showing *P*‐values ≤ .05 in each comparison. (I) Immunostaining of Otx2 (ectoderm marker), Sox17 (endoderm marker), Brachyury (mesoderm marker) and co‐staining with DAPI (blue) in EBs derived from hiPSCs‐SLE. Scale bar, 50 μm

Skin fibroblasts from the CNS‐SLE patient were transduced with Sendai virus (SeV) vectors (CytoTune, Life Technologies) encoding *OCT4*, *SOX2*, *KLF4* and *c‐MYC* at MOI of 5, for iPSCs generation. hiPSCs clones were picked and cultured on Matrigel‐coated plates and mTeSR1 medium. Subsequently, a characterization of three distinct hiPSCs clones was carried out. Using RT‐PCR, it was confirmed that clones were free from SeV vectors carrying transgenes detectable only in post‐transduction fibroblasts (ipF‐SLE) and not in the iPSCs at passage 20 (hiPSCs‐SLE) or in pre‐transduction fibroblasts (pF‐SLE) (Figure 1A). The reprogrammed hiPS cell line showed ESC‐like morphology and positivity for AP (Figure 1B), and the endogenous pluripotency markers including *OCT4*, *NANOG*, *SOX2* and *REX1* were reactivated in hiPSCs compared to fibroblasts (Figure 1C). Moreover, as confirmed by the PluriTest assay, high ‘pluripotency score’ and a low ‘novelty score’ revealed that the hiPSCs‐SLE expression profile is close to pluripotent stem cell signatures (Figure 1D). m‐FISH analysis of 70 metaphases for each sample ruled out any abnormality of karyotype (Figure 1E). At the protein level, the hiPSCs revealed positivity for the embryonic stem cell markers Nanog and Oct4, as demonstrated by immunofluorescence (Figure 1F). Moreover, embryo bodies derived from hiPSCs‐SLE were able to differentiate spontaneously towards the three germ layers as shown by RT‐qPCR for *GATA4* (endoderm), *HAND1* (mesoderm) and *PAX6* (ectoderm), and by immunofluorescence for Otx2 (ectoderm), Sox17 (endoderm) and Brachyury (mesoderm) (Figure 1G‐I).

### 3.2 mRNA expression profile analysis reveals Akt and Erk pathways involvement in SLE pathology

**Figure 2:**
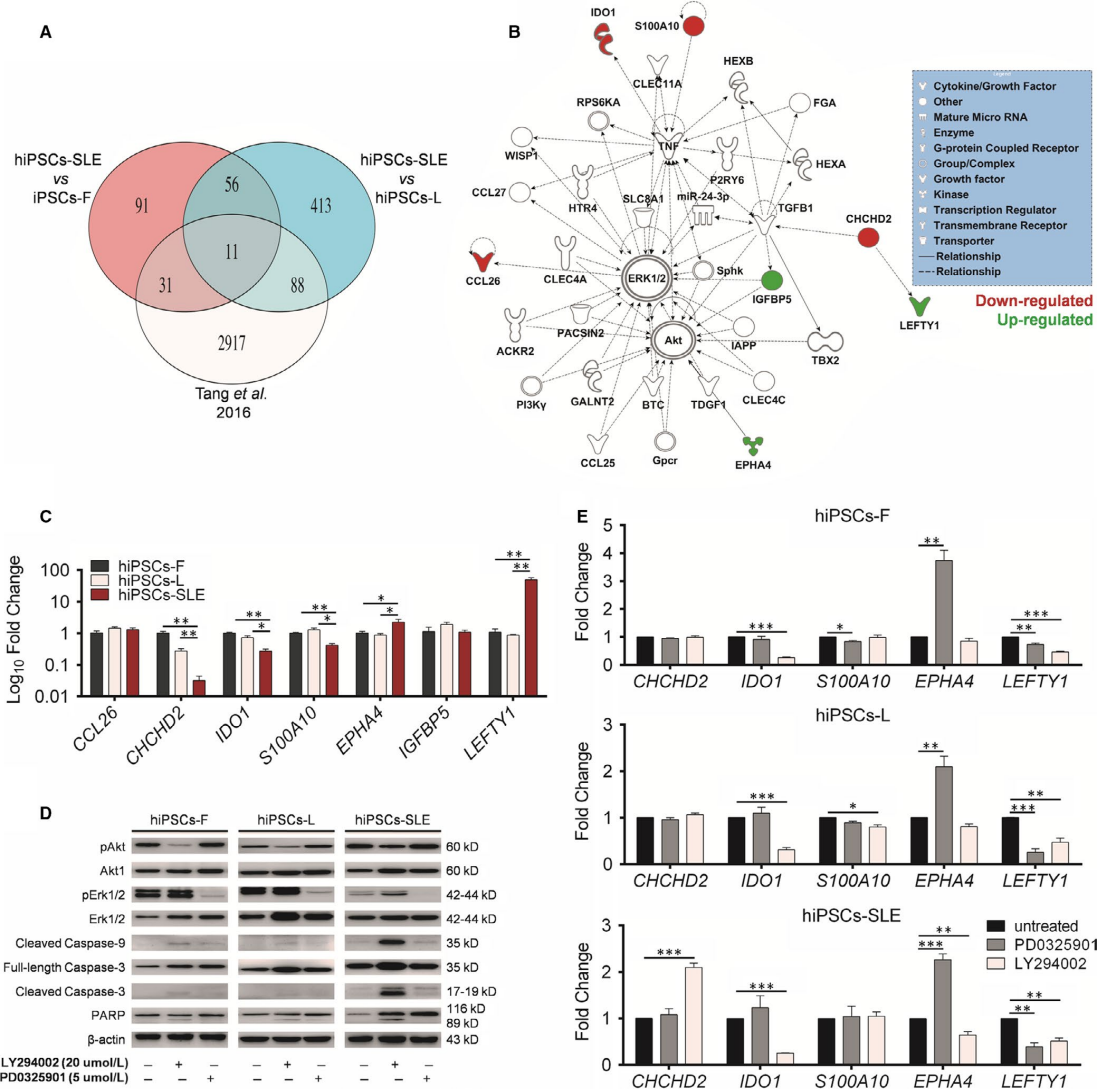
Identification of novel biomarkers correlated with SLE and involved in Erk and Akt pathways. (A) Venn diagrams showing the relative numbers of specific and common DEGs resulting from the hiPSCs‐CNS‐SLE (hiPSCs‐SLE vs hiPSCs‐F, hiPSCs‐SLE vs hiPSCs‐L) and hiPSCs‐SLE vs hiPSCs‐F reported in Tang et al 2016, according to an FDR corrected *P*‐value lower than 0.05. (B) IPA network of Akt‐Erk1/2‐related genes. Node colour represents the expression status based on our data, up‐regulated genes (green) and down‐regulated genes (red) in hiPSCs‐SLE. Each gene is displayed using various shapes that represent the functional class of the gene product, as indicated in the legend. (C) RT‐qPCR analysis validate the transcript levels of differential expressed genes and related to IPA analysis (*CCL26*, *CHCHD2*, *IDO1*, *S100A10*, *EPHA4*, *IGFBP5*, *LEFTY1)* in hiPSCs‐SLE compared to hiPSCs (‐F and ‐L). The transcript level was normalized against hiPSCs‐F. Two‐sided *t* test was used to compare hiPSCs‐SLE and hiPSCs‐F; hiPSCs‐SLE and hiPSCs‐L; *P*‐values ≤ .05*, <.01**. (D) The activity of Akt and Erk was verified by immunoblot analysis in untreated, LY294002 and PD0325901 conditions. Healthy hiPSCs (‐F and ‐L) and patient hiPSCs‐SLE exposed to LY294002 and PD0325901 suppress the phosphorylation of Akt (Ser473) and Erk (Thr202/Tyr204), respectively. The cleaved forms of Caspase‐3, Caspase‐9 and PARP were detected after treatment with LY294002 in all cell line, and in hiPSCs‐SLE untreated and treated with PD0325901. Full‐length blots/gels are presented in File S2. (E) After perturbation with LY294002 and PD0325901 the expression levels of *CHCHD2*, *IDO1*, *S100A10*, *EPHA4* and *LEFTY1* genes were assessed by RT‐qPCR. All expression values are relative to untreated hiPSCs and two‐sided *t* test was used to compare treated and untreated hiPSCs; *P*‐values ≤ .05*, <.01** and < .001***. (C, E) Data represent mean ± SEM of three biological replicates

To identify novel biomarker candidates potentially related to SLE, we performed mRNA profiling comparing hiPSCs‐SLE *vs*. hiPSCs‐F and hiPSC‐L. Expression values of mRNAs obtained were filtered for fold change (FC) greater than |1.5| and subjected to *t* test (*P‐*value cut‐off of 0.05) with Benjamini‐Hochberg (B‐H) FDR correction. Analysis of results allowed the identification of 189 and 568 DEGs in hiPSCs‐SLE compared to hiPSCs‐F and hiPSC‐L, respectively. Among them, 91 were expressed exclusively in hiPSCs‐SLE but not in hiPSCs‐F, 413 were present only in hiPSCs‐SLE but not in hiPSCs‐L, and 67 were found as exclusive genes of CNS‐SLE‐derived hiPSCs. To further strengthen our findings, we compared DEGs resulting from our analysis (CNS‐SLE patient‐derived hiPSCs) with those published in a recent work by Tang et al (SLE patient‐derived hiPSCs).^14^ Although the benefits of RNA‐seq over microarray in transcriptome profiling are well known, our analysis focused on 11 of 67 genes that resulted as common DEGs in CNS‐SLE and SLE in all the comparisons, as shown in Figure 2A. IPA revealed that 7 out of 11 genes are involved in Erk and Akt signalling (Figure 2B). Subsequently, these results were validated by RT‐qPCR, confirming the up‐regulation of *EPHA4* and *LEFTY1* and the down‐regulation of *CHCHD2*, *IDO1* and *S100A10* in hiPSCs‐SLE compared to healthy hiPSCs; the expression of *IGFBP5* and *CCL26* remained unchanged (Figure 2C).

Erk activity is suppressed in lupus T cells,^26^, ^27^ and the observation that T cells treated with Erk inhibitors can cause a lupus‐like disease in model systems, suggests that alterations in this pathway might induce the disease rather than being a consequence. It is known that the abnormal activation of Akt‐GSK3β signalling pathway increases proliferation rate of T lymphocytes of SLE patients.^28^ Enhanced activity of the PI3K signalling pathway with up‐regulation of phosphorylated Akt (pAkt) in T cells is also described in mouse models.^29^ We extended the investigation of the Erk and Akt pathways in hiPSCs‐SLE, by further characterizing these signalling pathways, with the aim to uncover new potential therapeutic targets. We confirmed by Western blot analysis a reduction of pErk and an induction of pAkt in hiPSCs‐SLE (Figure 2D), in agreement with previous reports on T cells of SLE patients.^26^, ^28^

Moreover, we perturbed Erk and Akt pathways in normal and SLE‐derived hiPSCs lines with the specific inhibitors PD0325901 and LY294002, respectively. The initial step was to confirm the reduction of pErk and pAkt in treated vs untreated hiPSC lines. Then, we evaluated the degree of activation of the apoptotic cascade through the detection of cleaved forms of Caspase‐3 (17‐19 kD), Caspase‐9 (35 kD) and PARP (89 kD). We identified the full‐length PARP protein (116 kD) in both untreated and treated hiPS cell lines. Interestingly, the active, cleaved forms of Caspase‐3 and Caspase‐9 and subsequently the inactive cleaved form of PARP were found in LY294002‐treated cell lines. Taking together, these results suggest that the activation of the apoptotic cascade is mediated by the inhibition of Akt pathway both in normal and diseased hiPSCs. Moreover, apoptosis signalling appears to be weakly activated also in hiPSCs‐SLE either in the presence or absence of the Erk inhibitor PD0325901, as demonstrated by a faint pro‐apoptotic signal of Caspase‐3 and Caspase‐9 (Figure 2D).

Next, expression levels of IPA‐selected candidate biomarkers (*S100A10, EPHA4, LEFTY1, IDO1* and *CHCHD2*) were investigated upon treatment with PD0325901 and LY294002. Although we could not find drastic changes in the expression of *S100A10* in the normal and diseased hiPSCs lines, all of the lines showed a marked up‐regulation of *EPHA4* and down‐regulation of *LEFTY1* after PD0325901 treatment, while a down‐regulation of *IDO1* and *LEFTY1* was detected after LY294002 treatment. Finally, hiPSCs‐SLE revealed an exclusive pattern represented by an over expression of *CHCHD2* and a reduction of *EPHA4* after exposure to LY294002.

### 3.3 The protective effects of ascorbic acid in hydrogen peroxide‐induced oxidative damage in pluripotent stem cells and their miRNAs profiling

Currently, a robust body of evidence suggests that the development of SLE has a strong genetic basis. Interplay between genetic susceptibility and environmental factors has been reported in SLE pathogenesis.^9^ In particular, ultraviolet light, diet, smoking, infections and other physiologic stressors, which stimulate ROS production and oxidative damage,^30^ may trigger, in genetically predisposed individuals, a cascade of events eventually leading to clinically symptomatic SLE. In this study, we selected H_2_O_2_ as an oxidative agent due its high production in SLE T cells.^31^ We next tested whether the antioxidant AA is able to counteract the oxidative stress effect on hiPSCs.^32^

**Figure 3:**
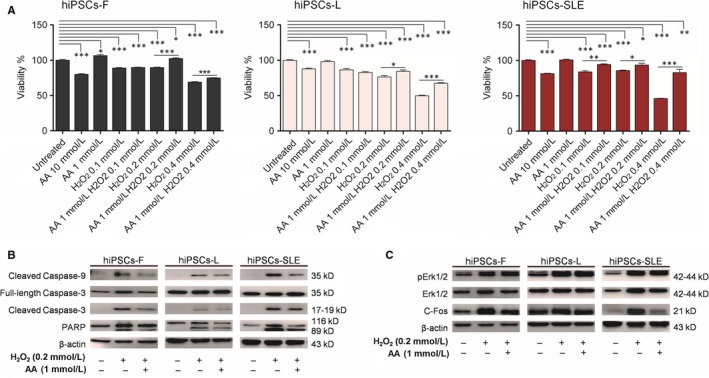
AA partially rescues H_2_O_2_‐treated hiPSCs. (A) Cell viability of hiPSCs exposed to different concentrations of H_2_O_2_ and AA was evaluated using an MTT assay. MTT quantification of antioxidant effects of AA was performed on hiPSCs pre‐treated for 24 h with or without 1 mmol/L AA under conditions of stress with 0.1, 0.2 and 0.4 mmol/L H_2_O_2_ for 4 h. Data represent mean ± SEM of three independent experiments. Results were analysed by two‐sided *t* test to compare each condition with relative untreated hiPSCs line or to compare pre‐treated AA condition plus H_2_O_2_ with relative H_2_O_2_ concentration in each hiPSCs line*, P*‐values ≤ .05*, <.01** and < .001***. (B) AA protects hiPSCs from H_2_O_2_‐induced apoptosis. hiPSCs were treated with 0.2 mmol/L H_2_O_2_ with or without 1 mmol/L AA pre‐treatment and cellular apoptosis was evaluated by Western blot. hiPSCs exposed to H_2_O_2_ result in an increased cleaved form of Caspase‐3, ‐9 and PARP levels while AA pre‐treatment on hiPSCs shows a reduction of cleaved form of Caspase‐3, ‐9 and PARP compared to H_2_O_2_ condition. In the same experimental conditions, we show a representative immunoblots of total Erk, pErk and c‐Fos (C) Erk pathway signalling results affected in hiPSCs treated with H_2_O_2_ showing increased pErk and c‐Fos levels, while following AA‐H_2_O_2_ condition pErk level remains increased and c‐Fos reduced. Full‐length blots/gels are presented in File S2

Cellular responses to H_2_O_2_ are known to differ in a concentration‐specific manner.^33^ Thus, in order to evaluate hiPSCs viability after treatment with H_2_O_2_ alone, with AA and their combination, we exposed normal and SLE‐derived hiPSCs to increasing concentrations of H_2_O_2_ (0.1, 0.2 and 0.4 mmol/L) for 4 hours, while two concentrations of AA (1 and 10 mmol/L) were selected following long‐term exposure (24 hours). Since 10 mmol/L AA determines 20% mortality while 1 mmol/L AA maintains 100% vitality, the latter condition was used to analyse its protective effect in the presence of different concentrations of H_2_O_2_. Cell viability, determined by MTT assay, weakly decreased at 0.1 and 0.2 mmol/L H_2_O_2_ (~10%‐25%) and sharply dropped at 0.4 mmol/L H_2_O_2_ (~30%‐55%) in all hiPSC lines. Combinations of 1 mmol/L AA with 0.2 and 0.4 mmol/L H_2_O_2_ showed an improvement by 10%‐20% viability in healthy control hiPSCs, and by 10%‐30% in hiPSCs‐SLE, confirming that AA plays a protective role in hiPSCs subjected to a short‐term oxidative stress. Then, we evaluated the putative activation of the apoptotic cascade after oxidative stress induced by 0.2 mmol/L H_2_O_2_, as well as the protective, anti‐apoptotic effect provided by AA. We confirmed that the cleaved forms of Caspase‐3, Caspase‐9 and PARP were increased in hiPSCs subjected to H_2_O_2_ compared to the untreated ones, while they were reduced in the presence of AA (Figure 3B)_._ Li et al have reported a marked decrease of the Erk pathway in T lymphocytes exposed to oxidizing agents through their inhibitory effects on protein kinase Cδ^34^; we performed a similar analysis in our experimental setting. Intriguingly, Western blotting revealed that H_2_O_2_ exposure and its combination with AA results in pErk and c‐Fos increase compared to untreated cells (Figure 3C), maybe due to a signalling pathway of H_2_O_2_‐induced Erk activation PKC‐independent, as previously demonstrated.^35^ Akt pathway results affected in hiPSCs treated with H_2_O_2_ showing increased pAkt levels, while, following AA‐H_2_O_2_ exposure, its levels were reduced compare to untreated and H_2_O_2_ conditions in both healthy and diseased lines (File S3).

A number of studies suggest that a specific set of miRNAs exerts a genetic and epigenetic control during SLE pathogenesis.^36^ In particular, the up‐regulation of miR‐21, miR‐29b, miR‐30a, miR‐126 and miR‐148a and the down‐regulation of miR‐142‐3p/5p and miR146a, positively correlate with DNA methylation changes occurring in CD4^+^ T cells, which are responsible for their marked autoreactivity, one of the hallmarks occurring in SLE.^37^

**Figure 4:**
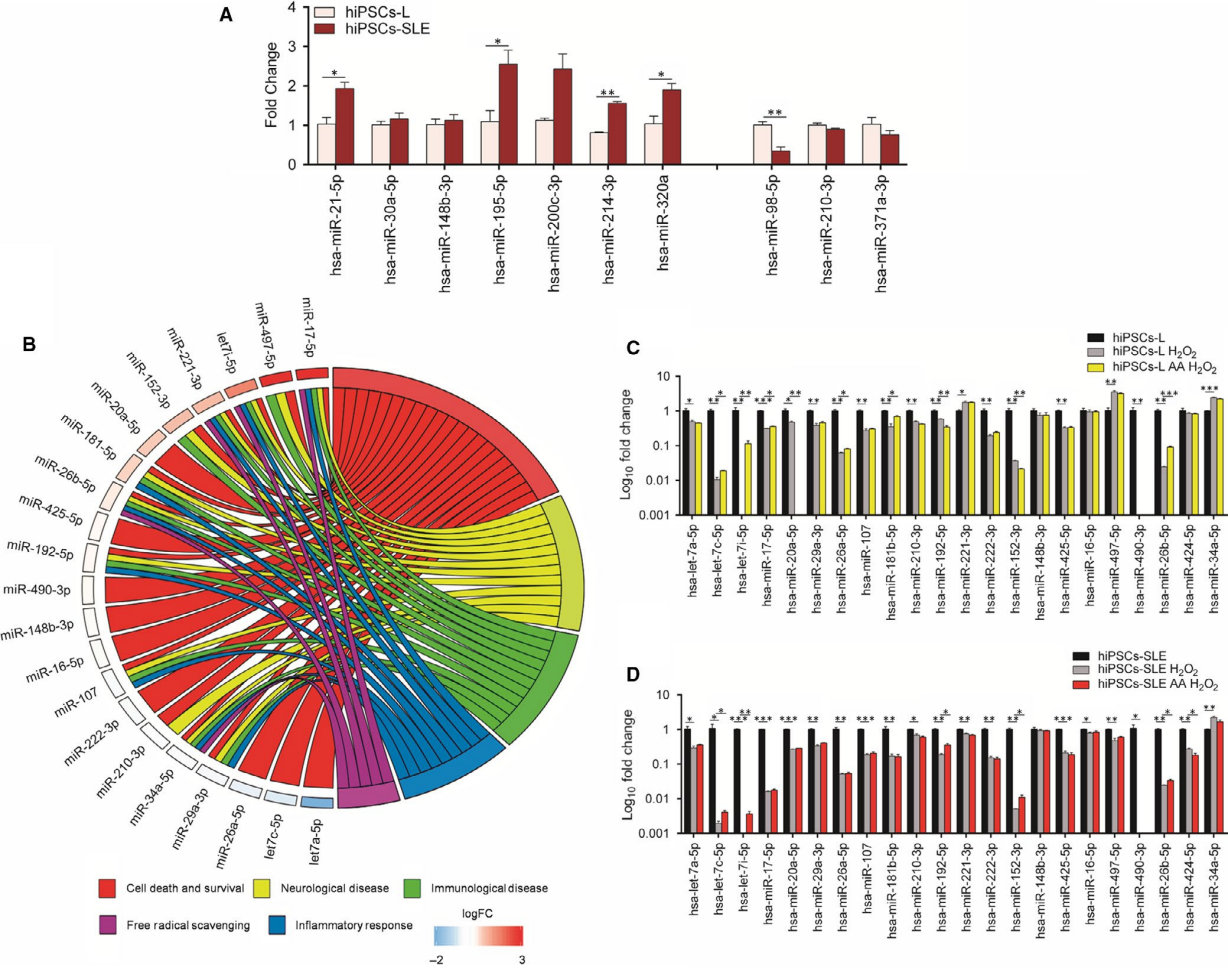
SLE and oxidative stress‐related miRNAs. (A) Up‐regulated and down‐regulated SLE‐associated miRNAs were validated by RT‐qPCR in hiPSCs‐SLE and hiPSCs‐L and all expression values are relative to hiPSCs‐L. A statistical comparison was performed between hiPSCs‐SLE and hiPSCs by two‐sided *t* test; *P*‐values ≤ .05*, <.01**. (B) Circos plots display the relationship between a list of selected miRNAs related to oxidative stress ordered by coloured rectangles representing the logFC (hiPSCs‐SLE vs hiPSCs‐L) and GO terms of relative miRNAs targets. The expression of oxidative stress‐related miRNAs was analysed by RT‐qPCR in hiPSCs‐L (C) and hiPSCs‐SLE (D) after 0.2 mmol/L H_2_O_2_ treatment and after treatment with 1 mmol/L AA for 24 h and then stimulated with 0.2 mmol/L H_2_O_2_ for 4 h. Data were normalized to miR‐103a and relative to hiPSCs (‐L and ‐SLE, respectively) maintained in mTeSR1 medium. A statistical differences between untreated and H_2_O_2_ conditions, H_2_O_2_ and AA‐H_2_O_2_ conditions in each hiPSCs line were performed by two‐sided *t* test; *P*‐values ≤ .05*, <.01** and < .001***. Quantitative data are showed as mean ± SEM of three biological replicates

In this work, we show a marked and significant up‐regulation of some of them, such as miR‐21, miR‐195, miR‐214 and miR‐320a, and a significant down‐regulation of miR‐98 in SLE‐derived hiPSCs compared to healthy hiPSCs‐L (Figure 4A), confirming the findings reviewed by Shen et al.^36^

Subsequently, we analysed a panel of oxidative stress‐responsive miRNAs, identifying 7 up‐regulated (miR‐17, miR‐497, let‐7i, miR‐221, miR‐152, miR‐20a, 181b), 4 down‐regulated (let‐7a, let‐7c, miR‐26a, miR‐29a) and 10 unchanged miRNAs in hiPSCs‐SLE compared to hiPSCs‐L. Next, IPA analysis was carried out considering categories related to SLE pathology, such as cell death and survival, free radical scavenging, inflammatory response, immunological and neurological diseases (Figure 4B).

While most of these findings are in agreement with previous work performed in different experimental settings, induction of miR‐497 and reduction of miR‐29a in hiPSCs‐SLE have never been linked to SLE. Contrasting results are those related to miR‐17 and miR‐20a, previously associated with SLE, but as down‐regulated miRNAs in SLE lymphocytes.^38^, ^39^

The same set of miRNAs was analysed in H_2_O_2_ and AA‐H_2_O_2_ conditions in healthy and diseased hiPSCs, normalizing data to the untreated ones. As expected, all the miRNAs, except miR‐148b and miR‐16, showed significant variations after H_2_O_2_ treatment in both cell lines. Ascorbic acid preconditioning produced an up‐regulation of let‐7c, let‐7i and miR‐26b in both cell lines. Moreover, under these conditions, only the hiPSCs‐L showed an induction of miR‐17, miR‐26a and miR‐181b, and a reduction of miR‐20a. Finally, we detected an opposite trend of expression for miR‐192 and miR‐152, down‐regulated in hiPSCs‐L and up‐regulated in hiPSCs‐SLE, while miR‐424 was found unaltered in healthy control and reduced in hiPSCs‐SLE (Figure 4C‐D).

## 4 DISCUSSION

Nowadays, the advances of iPSCs technology^40^ offer the opportunity to study in vitro autoimmune disease pathophysiology.^41^ Analogously, the fine dissection of network signalling and subsequent target identification approach appears promising in characterizing diseases.^42^

In this study, we took advantage of CNS‐SLE‐derived induced pluripotent stem cells to get further insights into the molecular mechanisms underlying SLE pathophysiology. To this end, we performed a comprehensive mRNA analysis of diseased and healthy (control) hiPSCs. Systematic comparison of the dataset, including those described by Tang et al, led to the identification of 11 genes differentially expressed in a consistent manner in CNS‐SLE and SLE patient‐derived hiPSCs compared to healthy hiPSCs. Of those, a sub‐cohort of 7 differentially expressed genes involved in the Erk and Akt pathways were exclusively inhibited or activated in hiPSCs‐SLE. More specifically, a down‐regulation of *CHCHD2*, *IDO1* and *S100A10* and an up‐regulation of *EPHA4* and *LEFTY1* were identified in diseased hiPSCs.

Chchd2 protein is a negative regulator of mitochondria‐mediated apoptosis, its down‐regulation increases the presence of Bax protein in the mitochondria‐enriched heavy membrane fraction, producing a strong apoptotic effect.^43^ The enzyme indoleamine 2,3‐dioxygenase 1 (*IDO1*) regulates immune responses to arrest inflammation and suppresses immunity through catabolism of tryptophan.^44^ In particular, *IDO1* dysregulation has been documented in patients with SLE.^45^ Thus, the low expression levels of *IDO1* in hiPSCs‐SLE could be a definite cause of an abnormal activation of the immune response. *S100A10* is a member of the S100 family of proteins containing two EF‐hand calcium‐binding motifs. S100A10 and Annexin A2, which is calcium‐regulated phospholipid‐binding protein, form plasminogen receptor.^46^ The up‐regulation of Annexin A2‐S100A10 heterotetramer causes increased fibrinolysis while the down‐regulation is reported in autoimmune conditions, as Annexin A2 is a target antigen for autoantibodies.^47^

The Eph receptor A4 (*EPHA4*), a member of the erythropoietin‐producing hepatocellular (Eph) family, promotes cell adhesion of multiple myeloma cells by enhancing the phosphorylation levels of Akt probably due to its interaction with CDK5.^48^ In our cellular system, the induction of *EPHA4* in the hiPSC‐SLE line might influence the increase of pAkt levels*. LEFTY1* gene encodes a secreted ligand of the TGF‐beta superfamily.^49^ Interestingly, inhibition of the Ras/Raf/Mek/Erk pathway dramatically enhances TGF‐mediated Lefty1 up‐regulation.^50^ Thus, the up‐regulation of *LEFTY1* could be explained by the low levels of pErk in the hiPSCs‐SLE. From these findings, it can be inferred that *EPHA4* and *LEFTY1* are associated with the development of SLE.

Erk and Akt pathways perturbation through the use of specific inhibitors, allowed us to confirm their modulatory role on the 5 candidates. Specifically, we found an up‐regulation of *CHCHD2* and a down‐regulation of *EPHA4* after treatment with the Akt signalling inhibitor LY294002. It should be noticed that, following the inhibition of Mek/Erk pathway with PD0325901, *LEFTY1* is reduced in all hiPSCs lines probably due to a failure in the activation of TGF‐β.

At the same time, we investigated the apoptotic cascade, demonstrating that the inhibition of the Akt pathway triggered an increase of apoptotic death in all cell lines, even though more pronounced in hiPSCs‐SLE compared to healthy control lines.

In T cells from patients with active lupus, it was demonstrated that oxidative damage inactivates PKCδ favouring Erk pathway inhibition. For instance, the reduced exposure to stressful environmental agents could delay the disease as well as the use of antioxidants could attenuate the ROS effects during inflammatory response reducing the flare severity.^51^ hiPSCs exposed to H_2_O_2_ show an increase in pErk and its c‐Fos target, both in diseased and healthy cells. The combined use of H_2_O_2_ and the antioxidant AA effectively reduces the activation levels of Caspase‐3, Caspase‐9 and PARP. In this latter condition, high pErk levels are preserved while the c‐Fos target gradually decreased.

On the basis of these findings, diseased and healthy control hiPSCs were used to discover new miRNAs associated with oxidative stress but not influenced by negative Erk regulation.

miRNAs are known to play a major role in biological and pathological processes, and their contribution in the regulation of target genes through degradation or repression in translation has been extensively investigated.^52^ The relative dysregulation of miRNAs associated to SLE pathology, such as miR‐21, miR‐195 and miR‐98, has been further confirmed in hiPSCs‐SLE. In this study, we compared oxidative stress‐related miRNAs expression in healthy and diseased cell lines, and Gene Ontology was performed by selecting categories compromised in the pathology, such as the inflammatory response, immune and neurological diseases. Our results indicate that the majority of up‐regulated miRNAs in hiPSCs‐SLE are involved in all selected categories with the exception of miR‐20a. It was reported that miR‐221 is a potential diagnostic biomarker of lupus nephritis,^53^ miR‐181a, miR‐152 and Let‐7i are increased in patients with SLE,^39^, ^54^, ^55^ while let‐7c, miR‐26a and let‐7a‐5p are decreased.^14^, ^56^, ^57^ Our data confirm previous findings, with the exception of miR‐17 and miR‐20a, whose expression levels increase, showing an opposite trend compared to the literature.^39^ We further extended miRNAs analysis, demonstrating a dysregulation of miR‐497 and miR‐29a, never associated so far to SLE. The up‐regulation of miR‐497 is involved in the negative modulation of Raf1 and Erk1 protein levels, but not in Erk2^58^ which could be a determining factor in the inhibition of the Erk pathway and therefore as intermediary of SLE progression. Furthermore, by comparing our data with miRNAs profile of Tang et al we found similar expression trend for miR‐21, let‐7c and let‐7a. In contrast, the miR‐17 and miR‐20a up‐regulation, and miR‐29a down‐regulation were only detected in our diseased cell line, while they were unaltered in Tang's data and therefore the different kind of expression might be considered as peculiar of CNS‐SLE pathology. Among the H_2_O_2_‐sensitive miRNAs, whose expression pattern shows opposite trend in healthy and diseased hiPSCs, miR‐424 is markedly reduced in hiPSCs‐SLE. Interestingly, this miRNA has been reported to protect neurons from oxidative stress by increasing the activity of Nrf2.^59^ Increased sensitivity to oxidative damage in hiPS‐SLE cells could be explained, at least in part, by the impairment of miR‐424 expression. The AA‐H_2_O_2_ condition highlights, in our experimental setting, a different response of the miR‐192 and miR‐152, which are up‐regulated in hiPSCs‐SLE and down‐regulated in healthy control hiPSCs, respectively. The down‐regulation of miR‐192 has a protective effects in hepatocytes, limiting liver injury caused by oxidative stress.^60^ Analogously, we hypothesize a similar biological meaning for miR‐424 and miR‐192. Overexpression of miR‐152 suppresses cell proliferation and invasion of non‐small‐cell lung cancer by inhibiting FGF2.^61^ Considering the up‐regulation of miR‐152 in hiPSCs‐SLE, it would be interesting to investigate whether the AA‐H_2_O_2_ condition may suppress their proliferative potential through a similar mechanism previously described by Cheng et al.

In this work, we provide evidence that the integration of different gene expression profile datasets is a powerful and useful tool to better dissect biological networks related to SLE. Based on the identification of the SLE biomarker candidates, we propose that the findings of this study might contribute to get additional insights in the diagnosis or potential therapeutic applications for the treatment of SLE pathology.

## CONFLICTS OF INTEREST

The authors confirm that there are no conflicts of interest.

## AUTHORS' CONTRIBUTIONS

MTDA, GS and GC: contributed in the conception and design of the experiments, and manuscript writing. GS: performed the bioinformatics analysis. MTDA, EIP, SS and MLC: performed the experiments and analysed the data. CC and AS performed and analysed m‐FISH. MTDA and GS: interpreted the data and prepared figures. SG, EF and UA: recruited patients and supervised clinical aspects. All authors revised critically the manuscript and approved the final version to be published.

## ETHICS APPROVAL AND CONSENT TO PARTICIPATE

The ‘Magna Graecia’ University of Catanzaro and the Azienda Ospedaliero—Universitaria ‘Mater Domini’ approved the study and confirmed that all experiments and methods were carried out according to the World Medical Association Declaration of Helsinki. All study participants provided written informed consent.

## Data Availability

The processed and normalized datasets supporting the conclusions of this article are included within the article (File S1). Raw data used during the current study are available from the corresponding author upon reasonable request.
