# Supplementary material for: Establishment and characterization of induced pluripotent stem cells (iPSCs) from central nervous system lupus erythematosus
Source: J Cell Mol Med. 2019 Sep 19;23(11):7382–94. doi: 10.1111/jcmm.14598 (PMC6815917; doi:10.1111/jcmm.14598)
Supplement: Supplementary file 1 [file JCMM-23-7382-s001.docx]

**Supplementary file 1**

Differential expressed genes.

hiPSCs-SLE_vs_hiPSCs-F, hiPSCs-SLE_vs_hiPSCs-L.

**Supplementary file 2**

Full‐length gels and blots in the main article.

Each Western blot filter contains three biological replicates and the red brackets show the biological replicate represented in the main figures 2 and 3.

**Supplementary file 3**

The Akt activity was verified by immunoblot analysis in hiPSCs treated with 0.2 mM H_2_O_2_ with or without 1mM AA pre-treatment.

Healthy hiPSCs (-F and -L) and patient hiPSCs-SLE exposed to H_2_O_2_ show an increase of phosphorylated Akt (Ser473), instead restored when pre-treated with AA. The pAkt level variation was comparable in the three cell lines.
