# Supplementary material for: Establishment and characterization of induced pluripotent stem cells (iPSCs) from central nervous system lupus erythematosus
Source: J Cell Mol Med. 2019 Sep 19;23(11):7382–94. doi: 10.1111/jcmm.14598 (PMC6815917; doi:10.1111/jcmm.14598)

Supplementary file 2. Full-length gels and blots in the main article

Each western blot filter contains three biological replicates and the red brackets show the biological replicate represented in the main figures 2 and 3.

Figure 1a

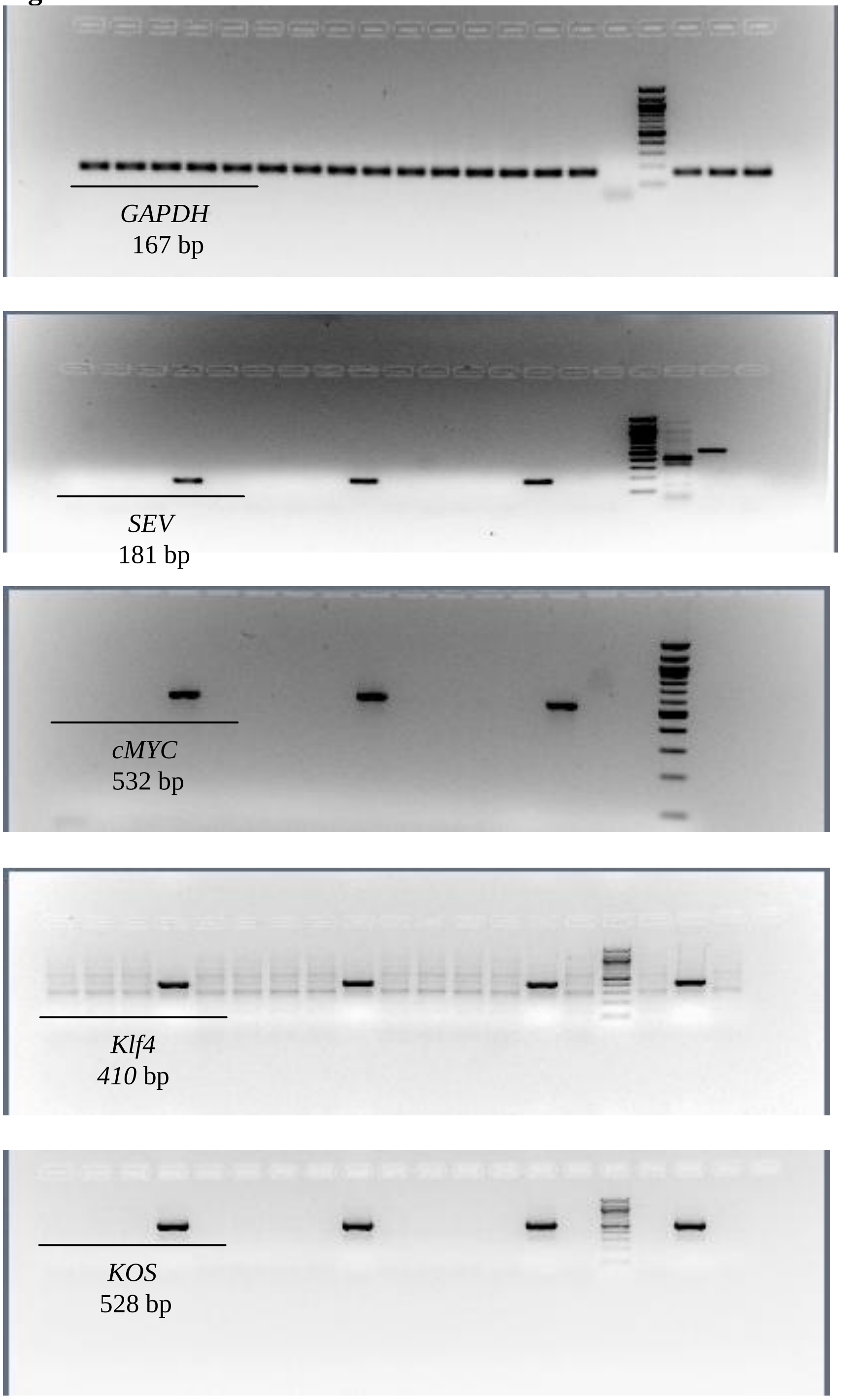

Figure 2b

<u>hiPSCs-F</u>

pAkt, 60 kDa

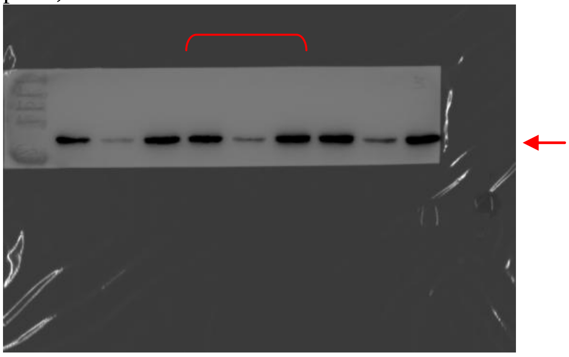

Akt1, 60 kDa

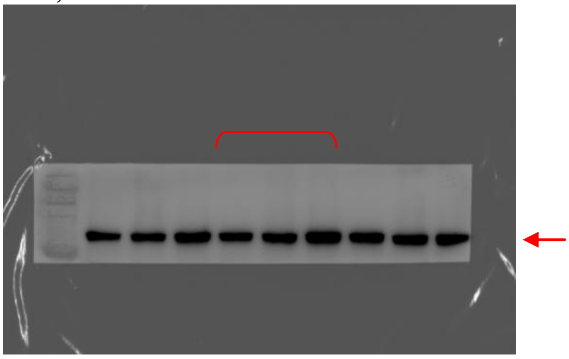

pErk1/2, 42-44 kDa

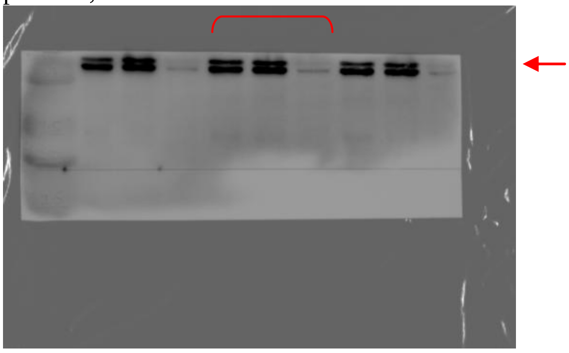

Erk1/2, 42-44 kDa

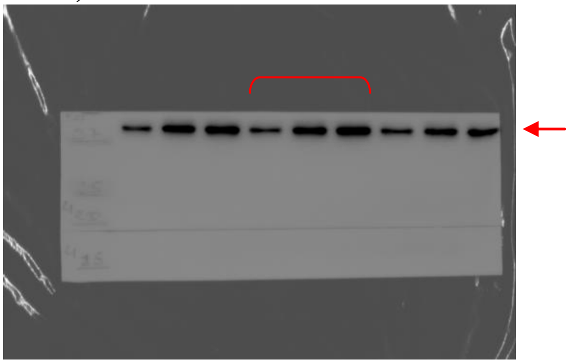

Cleaved Caspase-9, 35 kDa

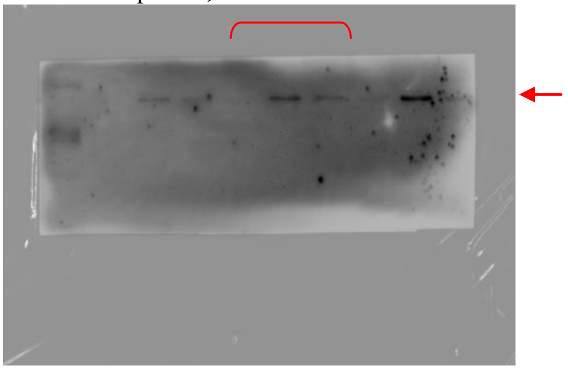

Full-length Caspase-3, 35 kDa

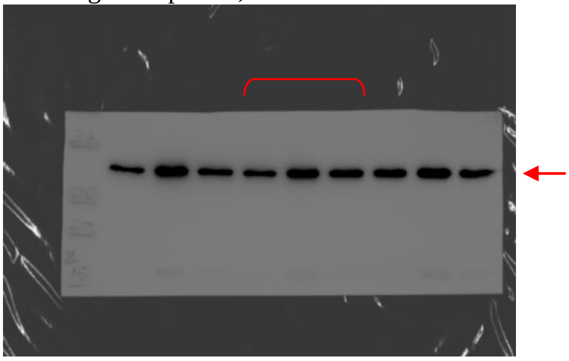

Cleaved Caspase-3, 17-19 kDa

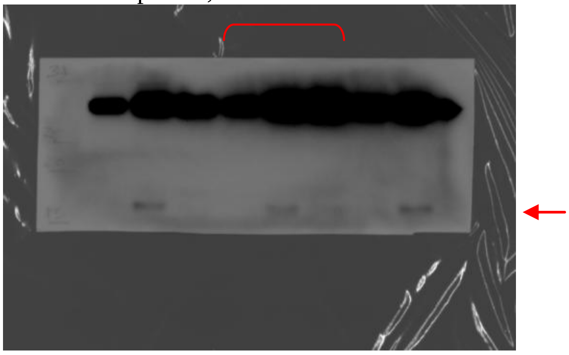

PARP, 89-116 kDa

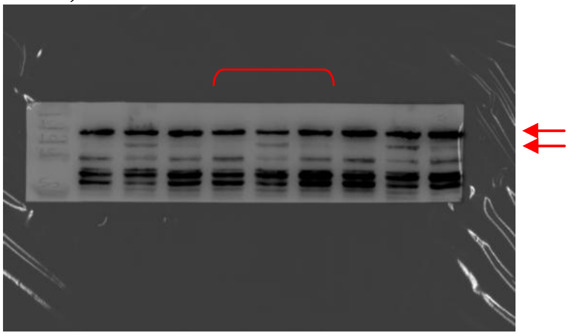

β-actin, 43 kDa

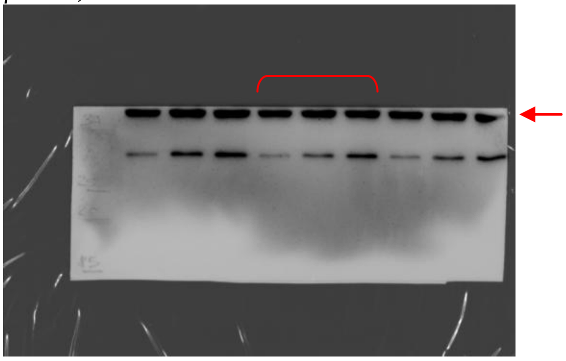

<u>hiPSCs-L</u>

pAkt, 60 kDa

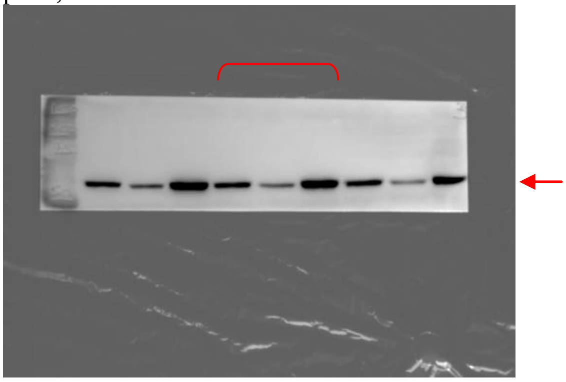

Akt1, 60 kDa

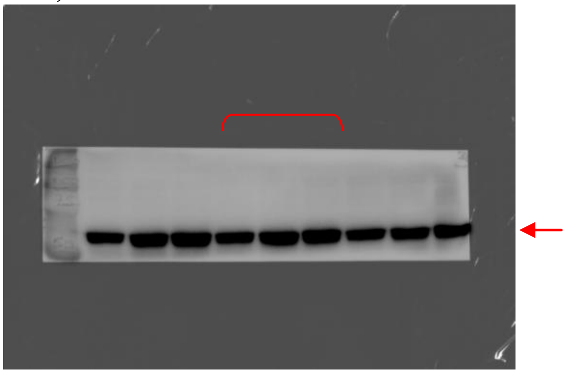

pErk1/2, 42-44 kDa

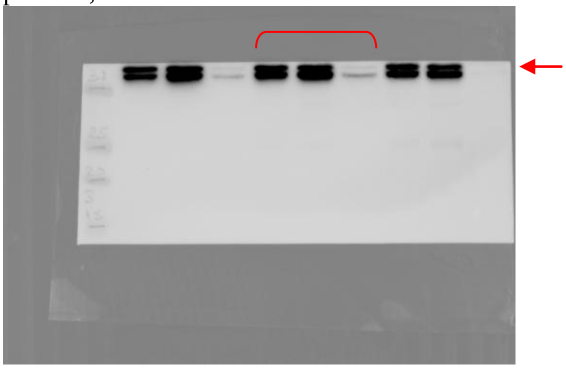

Erk1/2, 42-44 kDa

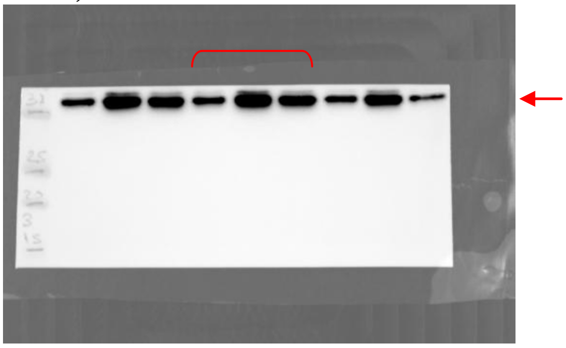

Cleaved Caspase-9, 35 kDa

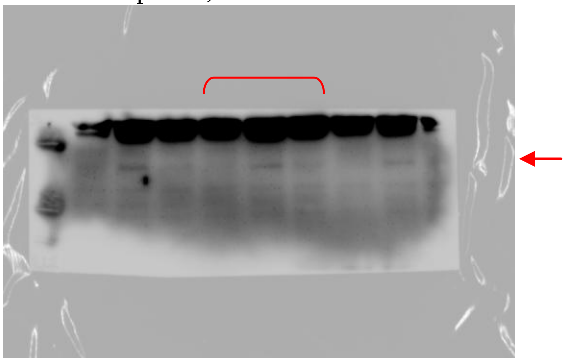

Full-length Caspase-3, 35 kDa

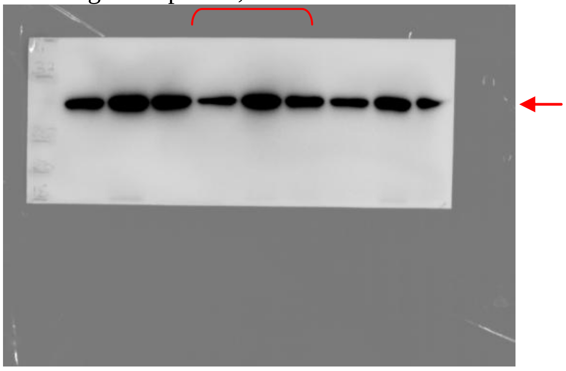

Cleaved Caspase-3, 17-19 kDa

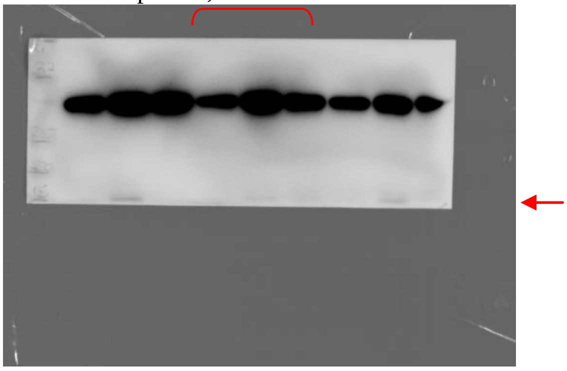

PARP, 89-116 kDa

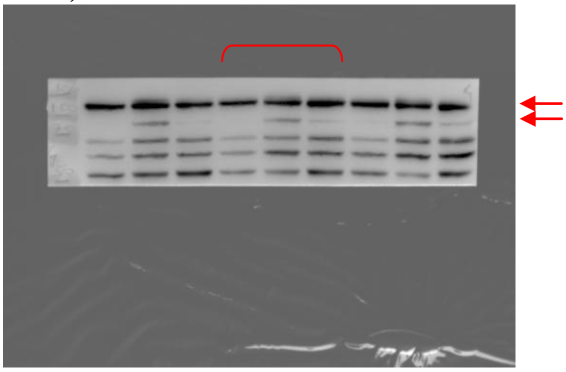

β-actin, 43 kDa

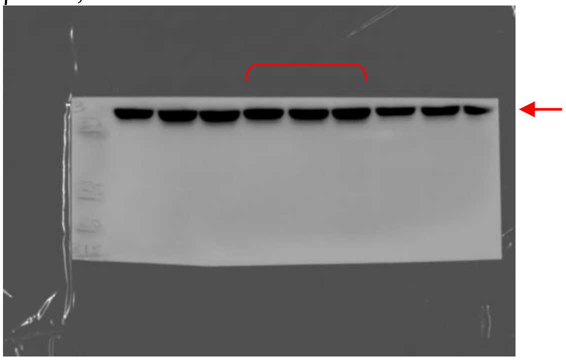

<u>hiPSCs-SLE</u>

pAkt, 60 kDa

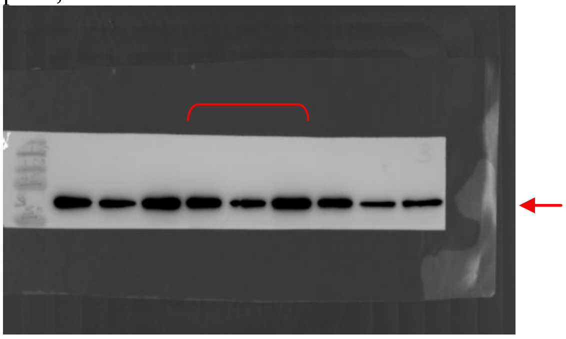

Akt1, 60 kDa

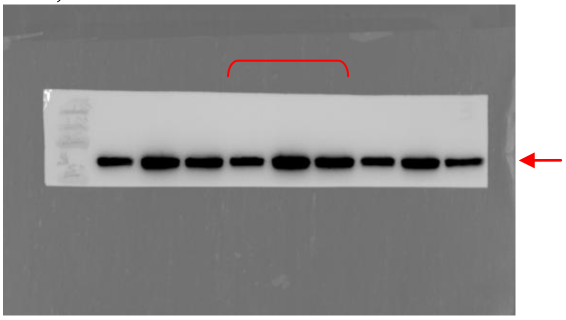

pErk1/2, 42-44 kDa

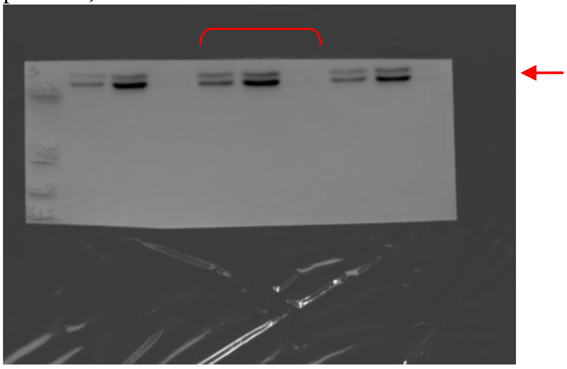

Erk1/2, 42-44 kDa

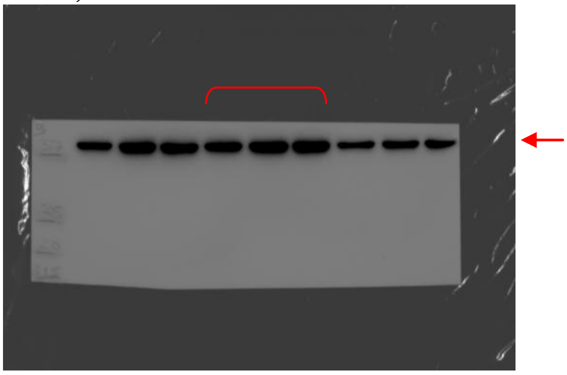

Cleaved Caspase-9, 35 kDa

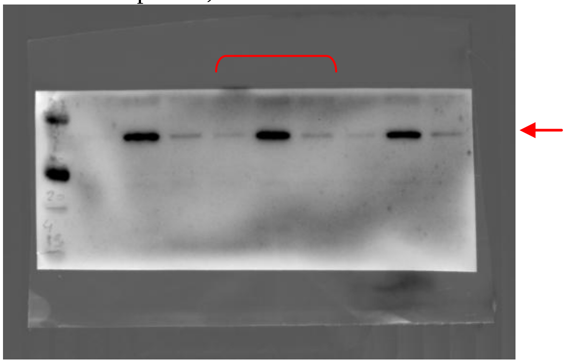

Full-length Caspase-3, 35 kDa

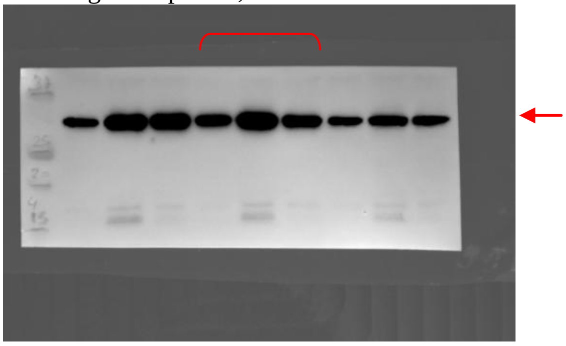

Cleaved Caspase-3, 17-19 kDa

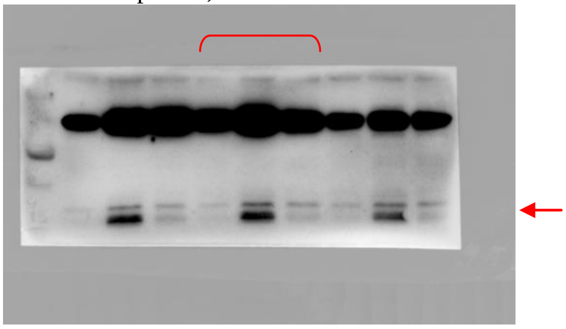

PARP, 89-116 kDa

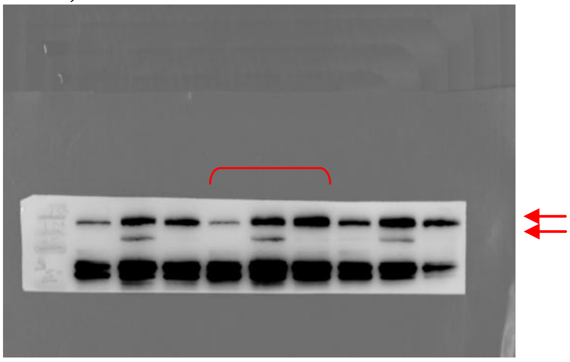

β-actin, 43 kDa

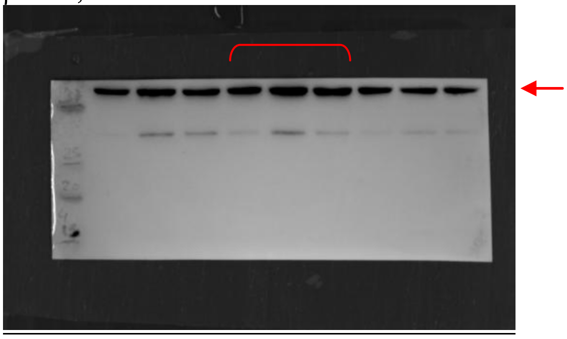

Figure 3b

<u>hiPSCs-F</u>

Cleaved Caspase-9, 35 kDa

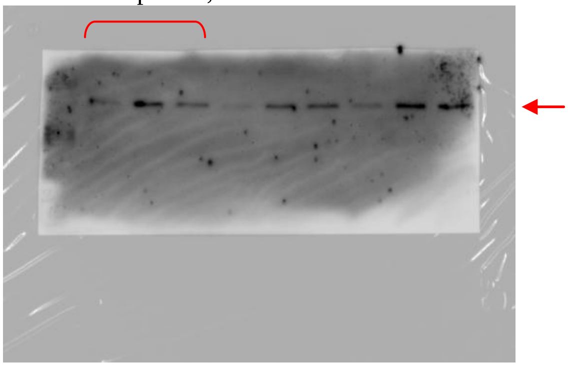

Full-length Caspase-3, 35 kDa

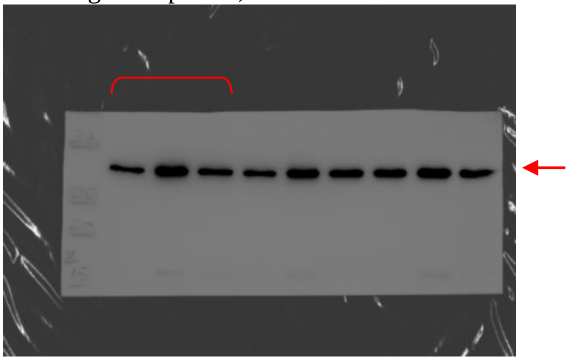

Cleaved Caspase-3, 17-19 kDa

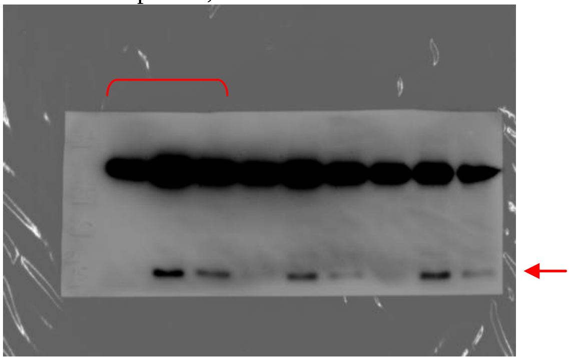

PARP, 89-116 kDa

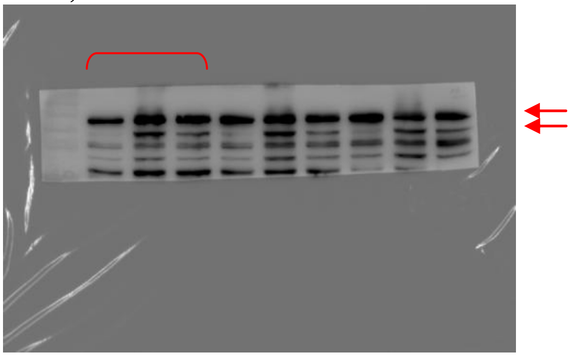

β-actin, 43 kDa

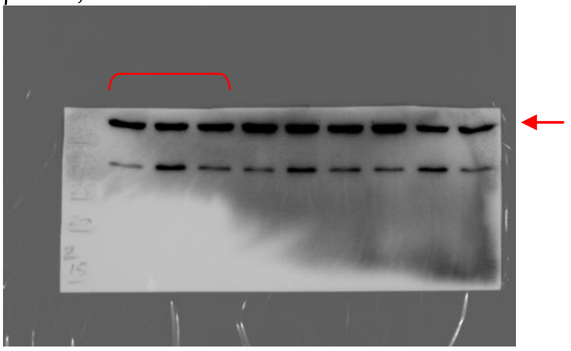

hiPSCs-L

Cleaved Caspase-9, 35 kDa

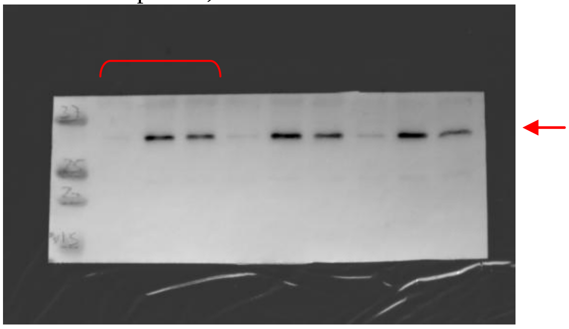

Full-length Caspase-3, 35 kDa

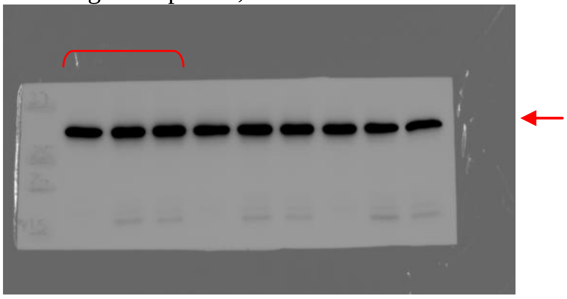

Cleaved Caspase-3, 17-19 kDa

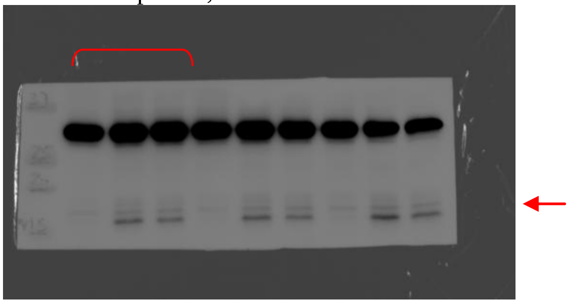

PARP, 89-116 kDa

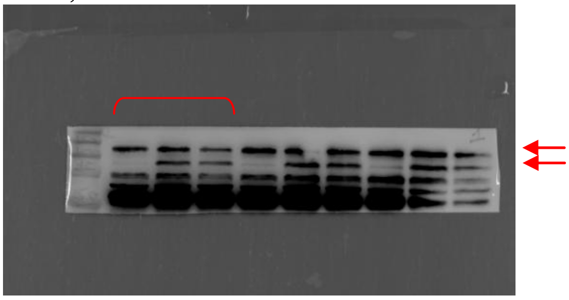

β-actin, 43 kDa

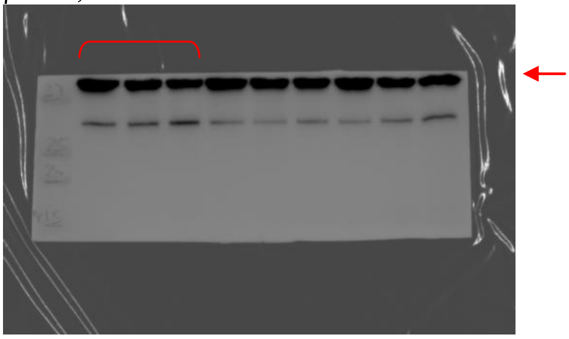

hiPSCs-SLE

Cleaved Caspase-9, 35 kDa

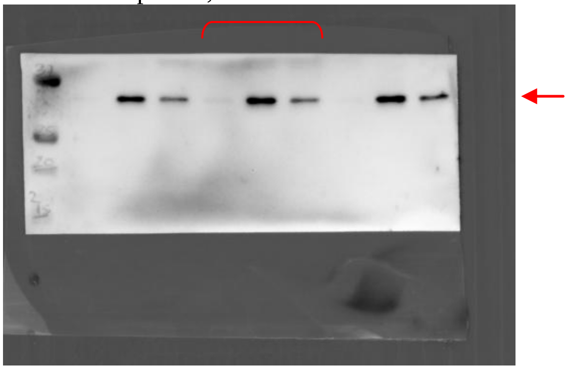

Full-length Caspase-3, 35 kDa

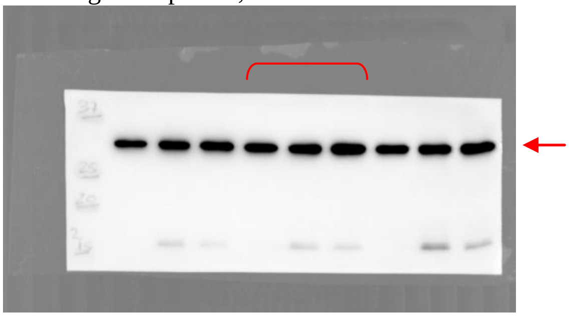

Cleaved Caspase-3, 17-19 kDa

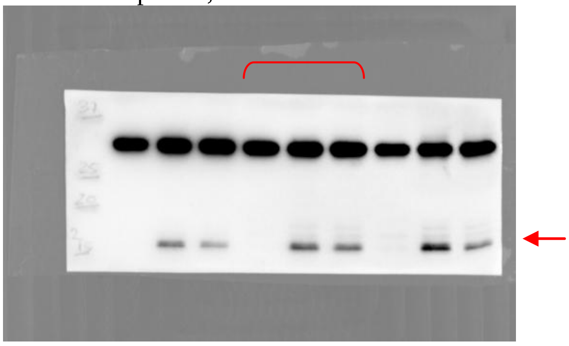

PARP, 89-116 kDa

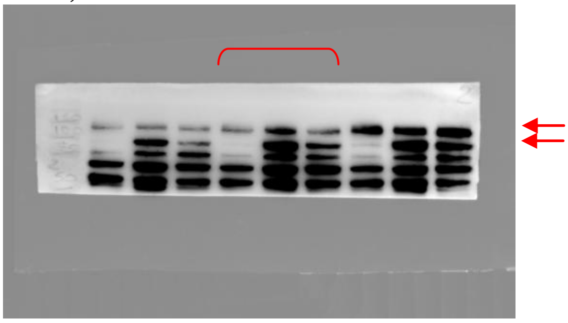

β-actin, 43 kDa

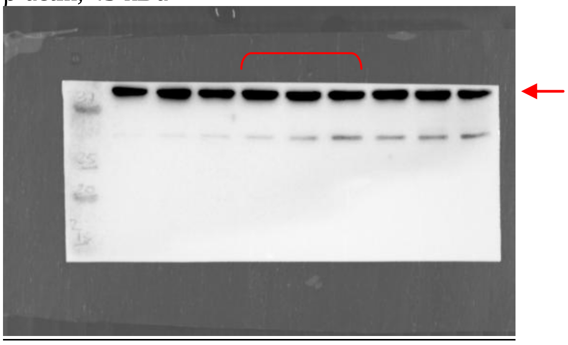

Figure 3c

<u>hiPSCs-F</u>

pErk1/2, 42-44 kDa

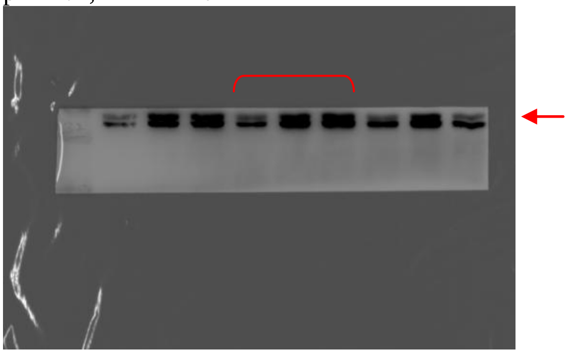

Erk1/2, 42-44 kDa

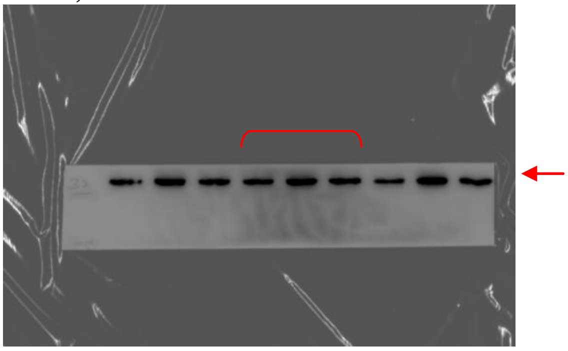

C-Fos, 21 kDa

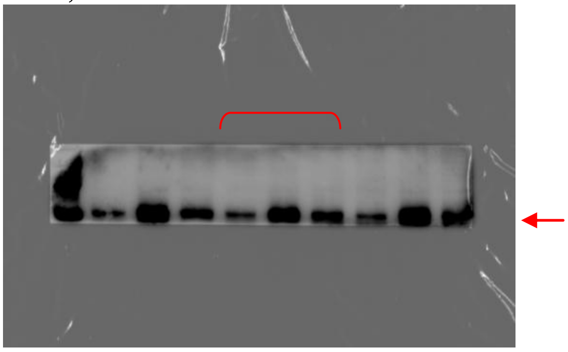

β-actin, 43 kDa

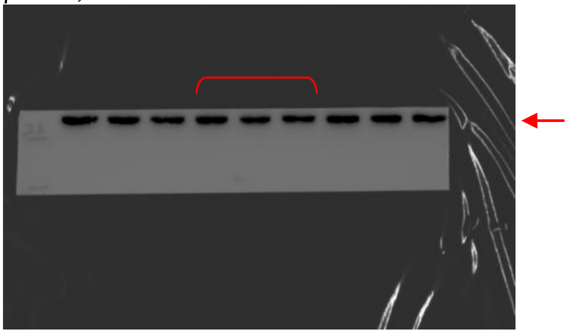

hiPSCs-L

pErk1/2, 42-44 kDa

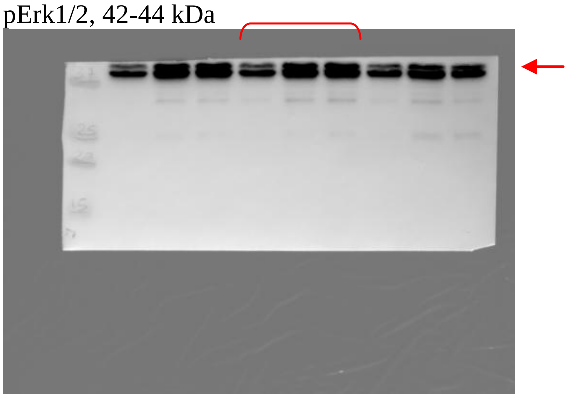

Erk1/2, 42-44 kDa

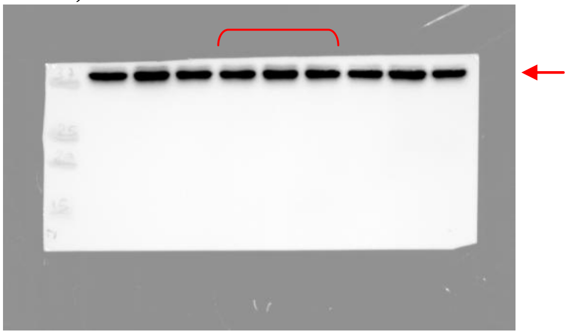

C-Fos, 21 kDa

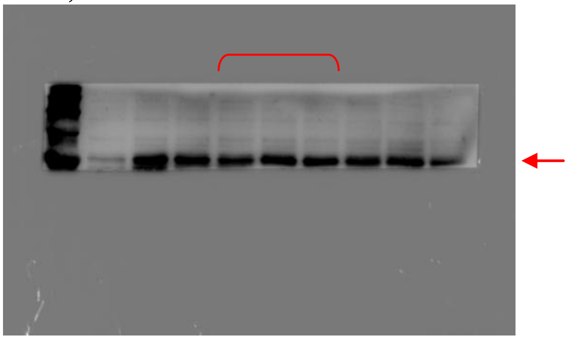

β-actin, 43 kDa

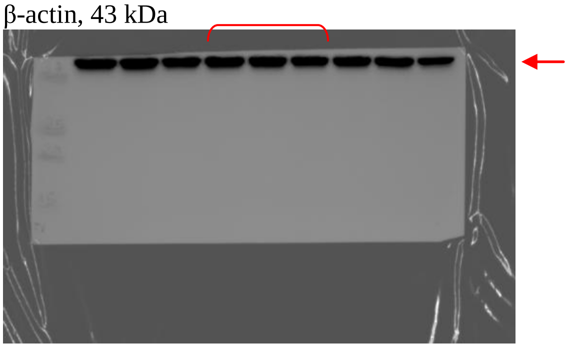

<u>hiPSCs-SLE</u>

pErk1/2, 42-44 kDa

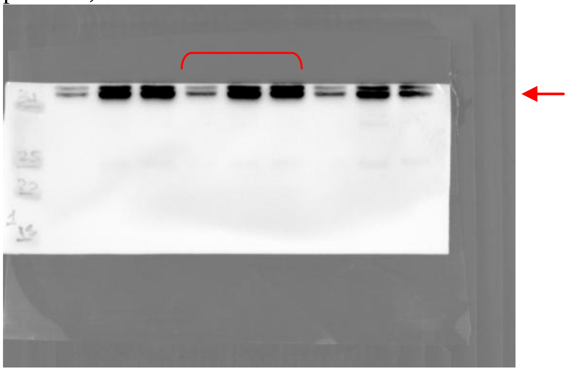

Erk1/2, 42-44 kDa

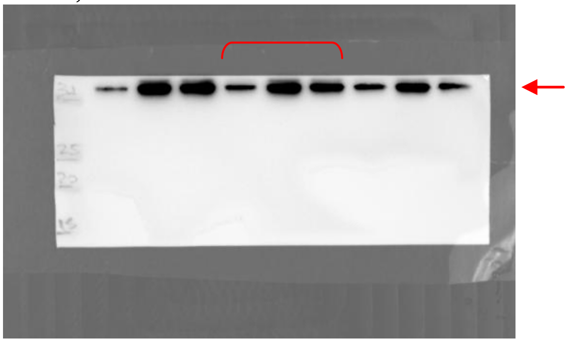

C-Fos, 21 kDa

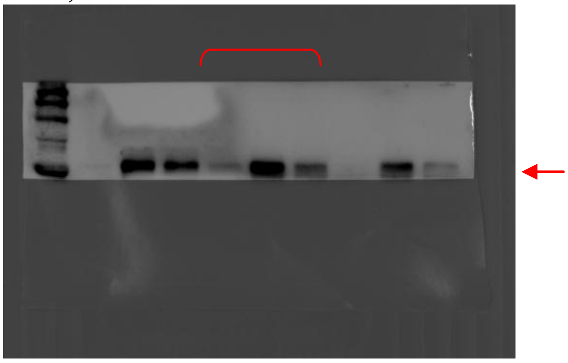

β-actin, 43 kDa

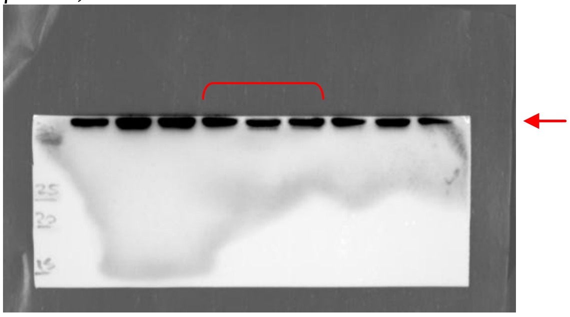

Supplement: Supplementary file 2 [file JCMM-23-7382-s002.pdf]
