# Supplementary figures and images for: Establishment and characterization of induced pluripotent stem cells (iPSCs) from central nervous system lupus erythematosus
Source: J Cell Mol Med. 2019 Sep 19;23(11):7382–94. doi: 10.1111/jcmm.14598 (PMC6815917; doi:10.1111/jcmm.14598)

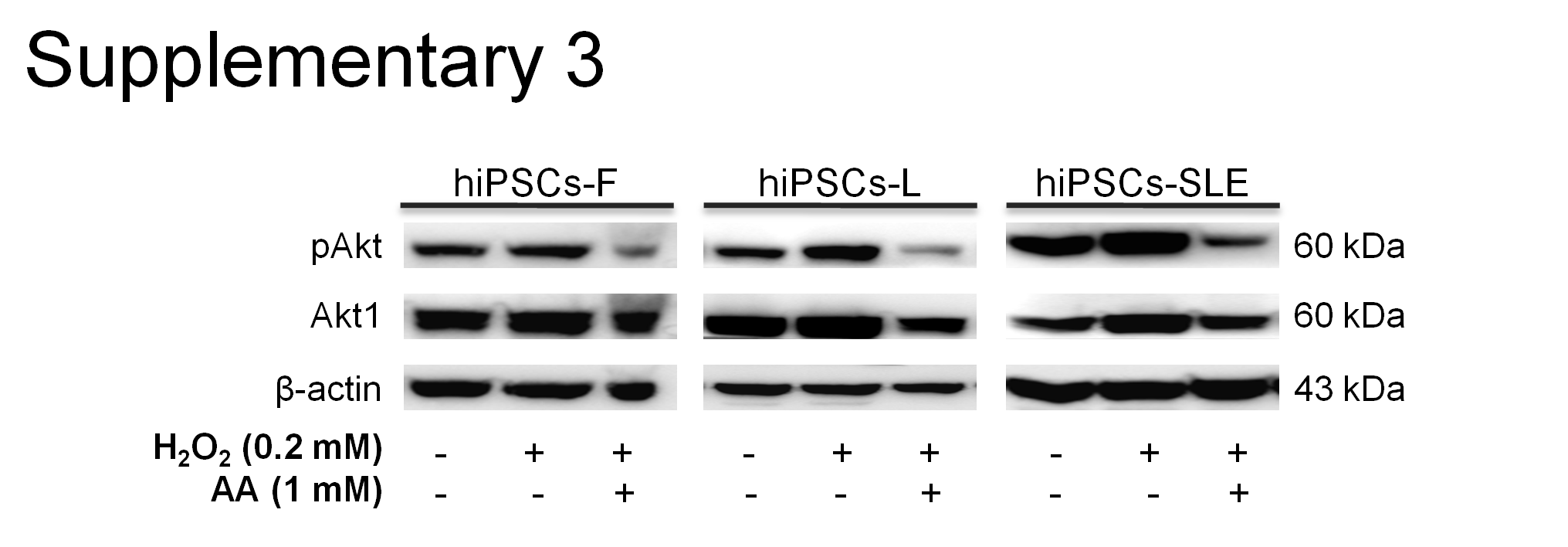

Supplement: Supplementary file 3 [file JCMM-23-7382-s003.tif]
